# Oral Allergy Syndrome in a Child Provoked by Royal Jelly

**DOI:** 10.1155/2014/941248

**Published:** 2014-03-31

**Authors:** Fantini Paola, Delle Donne Pantalea, Calogiuri Gianfranco, Ferrannini Antonio, Vacca Angelo, Nettis Eustachio, Di Leo Elisabetta

**Affiliations:** ^1^Section of Allergy and Clinical Immunology, Department of Internal Medicine and Infectious Diseases, University of Bari Medical School, Policlinico, Piazza Giulio Cesare, 11 70124 Bari, Italy; ^2^Fourth Pneumology Department, Pneumologic Hospital A. Galateo, San Cesario di Lecce, Italy; ^3^Department of Internal Medicine and Clinical Oncology, University of Bari Medical School, Policlinico, Piazza Giulio Cesare, 11 70124 Bari, Italy; ^4^Section of Allergy and Clinical Immunology, Unit of Internal Medicine—“F. Miulli” Hospital, Acquaviva delle Fonti, Bari, Italy

## Abstract

Royal jelly has been demonstrated to have several physiological activities. However, in the literature, different reactions induced by royal jelly are reported. We describe a case of seven-year-old child that was referred to our observation for two episodes of oral allergy syndrome (OAS) that appeared ten minutes after ingestion of royal jelly. Skin prick test with standard panel of inhalant and food allergens, a prick-to-prick test using the royal jelly's extract responsible for patient's reactions, and royal jelly patch test with extemporaneous preparation were performed. The specific IgE by ImmunoCAP System method versus Hymenoptera venom, inhalant allergens, food allergens, and lipid transfer proteins was dosed. According to the positive reactions to royal jelly both by prick-by-prick test and by a first reading patch test, royal jelly immediate hypersensitivity was diagnosed. According to the positive response for almond in both *in vivo* and *in vitro* tests we can think of the royal jelly contamination with almond pollen as possible cause of patient's reaction. Moreover, from the results of specific IgE titers versus Compositae pollens, we have argued the possibility that this case of royal jelly allergy could be explained also by the mechanism of cross-reaction with Compositae pollens.

## 1. Introduction

Royal jelly is a creamy, yellow-white, acidic material. It is a secretion produced by the hypopharyngeal and mandible glands of worker honey bees (*Apis mellifere*) [1]. It contains many important compounds with biological activity, such as free amino acids, fatty acids, sugars, minerals, proteins, and vitamins [2, 3]; approximately half of its dry weight consists of protein.

Royal jelly has been demonstrated to have several physiological activities ranging from promoting growth in children to improving general health status and enhancing longevity in the elderly [4].

In the literature, anaphylactic reaction [1, 5–7], death [8], acute asthma [9–12], and contact dermatitis [13] induced by royal jelly are reported.

## 2. Case Report

We describe a case of seven-year-old child that was referred to our observation for two episodes of oral allergy syndrome (OAS). The two episodes were exactly alike. Royal jelly was prescribed to patient by her physician in order to promote her growth. Ten minutes after ingestion her mother reported the onset of typical oropharyngeal symptoms of the OAS such as lip, tongue edema, and pa late itch. In both episodes the symptoms were treated with oral antihistamines with complete disappearance of them. No previous food and inhalants sensitization, contact and atopic dermatitis, adverse drug reaction (ADR) resulted in patient's clinical history. Skin prick test (SPT) was performed with standard panel of inhalant and food allergens. Positive reactions were observed to cat dander and grass, olive, cypress, and* Parietaria* pollen, and, among food allergens, positive response was obtained only for almond. We dosed the specific IgE by ImmunoCAP System method [14–17] versus hymenoptera venom (honey bee, wasp, yellow jacket, bumblebee, white-faced hornet), inhalant allergens (Compositae, Cupressaceae, Gramineae, Oleaceae, and Urticaceae), food allergens (nuts and seeds, peach, apple, egg white, and yolk), profilins (Bet v2, Pru p1, and Pru p4), and lipid transfer proteins (Pru p3, Ara h9, and Cor a8). Values higher than 0.10 (KUA/L) of specific IgE indicate sensitization to allergen according to the manufacturer (Phadia AB, Uppsala, Sweden, now Thermo Fisher Scientific).

## 3. Results

The patient showed higher levels of specific IgE than the normal range against honey bee, yellow jacket, white-faced hornet, and bumblebee with a range between 0.12 and 0.19 KUA/L, other positive responses were found for* Lolium perenne *(3.40 KUA/L),* Cynodon dactylon *(0.34 KUA/L),* Cupressus sempervirens *(5.30 KUA/L),* Parietaria judaica *(11.3 KUA/L),* Artemisia vulgaris *(0.25 KUA/L),* Taraxacum officinalis* (0.22 KUA/L),* Matricaria chamomilla *(0.29 KUA/L),* Chrysanthemum *(0.34 KUA/L), almond (1.17 KUA/L), peanut (0.60 KUA/L), hazelnut (0.18 KUA/L), soybean (0.51 KUA/L), sunflower seeds (0.18 KUA/L), peach (0.27 KUA/L), apple (0.88 KUA/L), and Pru p 3 (0.26 KUA/L).

Really, SPT positivity and specific serum IgE that were found were not clinically significant since our patient had not related symptoms, but they revealed an atopic status.

A prick-to-prick test using the royal jelly's extract responsible for patient's reactions gave a positive result with a wheal diameter of 6 mm.

The patient was patch tested with extemporaneous preparation with royal jelly undiluted. The patch test occlusion time and its reading followed the revised European task force on atopic dermatitis recommendations [18]. A first reading of royal jelly patch test (done 20 minutes after the application of allergens) gave an immediate positive result. So, according to the positive reactions to royal jelly both by prick-by-prick test and by patch test, royal jelly immediate hypersensitivity was diagnosed.

## 4. Discussion

As the symptoms appeared at the time of patient's first intake, we assumed that she had been sensitized for royal jelly previously or her symptoms were induced by the cross-reactivity between royal jelly and other common environmental allergens such as bee, honey, and pollens. It is known that pollination can be accomplished by cross-pollination or by self-pollination [19]. Pollen vectors can be animals, usually insects, including Hymenoptera. These pollinators can be wild such as the* Bumblebees* [20] or bred by human being such as honey bees. For the bees, pollen is an important source of proteins. Therefore, pollen is included in the royal jelly's composition distributed to all bees' larvae in the colony. A great amount of pollens which contaminate royal jelly are transferred in beehives with the nectar where pollen grains fall because of the wind or of visitor insect.

Pollen which adheres to bee's body can also fall in not yet percolated cells of the honeycomb. Farther, young bees take part in process of royal jelly and honey manufacturing; in this way, some ingested pollen grains end up in the harvest. Many fruit-bearing plants benefit by entomophilous pollination, including almond. In fact, the almonds are often placed near beehives. According to the positive response for almond in both* in vivo *and* in vitro *tests, we can think of the royal jelly contamination with almond pollen as possible cause of patient's reaction. Moreover, from the results of specific IgE titers we suspected the coexistence of* Compositae *sensitization, since specific IgE to* Taraxacum officinale, Matricaria chamomilla, Artemisia vulgaris, *and* Leucanthemum vulgare *was found. So, we have argued the possibility that this case of royal jelly allergy could be explained also by the mechanism of cross-reaction with Compos itae pollens.

In previous years, allergic reactions to bee products, including royal jelly, have been reported in subjects also allergic to Compos itae plant family [21], but this is the first report of OAS based on an immediate IgE mediated reaction confirmed by prick-by-prick and patch test carried out with royal jelly.

Almost all patients who have had allergic reactions to royal jelly had a history of asthma, allergic rhinitis, eczema, or atopy just as our patient [5, 11, 12]. Therefore, atopic individuals might have an increased risk of sensitization to royal jelly and to develop allergic reactions to royal jelly [1, 8] than those who are not atopic.

There seems to be a significant association between positive royal jelly skin test and atopy, adverse reactions to royal jelly and a history of sensitivity to other allergens.

Nevertheless, individuals with a history of asthma, atopy, or any other allergic problems should be cautioned against taking royal jelly products and at the same time their physicians should be aware of the possibility of severe allergic reactions caused by royal jelly in these particular patients.
